# The function of the south-Levantine Late Chalcolithic and Early Bronze Age basalt vessels bearing circumferential depressions: Insights from use-wear analyses

**DOI:** 10.1371/journal.pone.0252535

**Published:** 2021-06-04

**Authors:** Karolina Hruby, Marzena Cendrowska, Rivka Chasan, Iris Groman-Yaroslavski, Danny Rosenberg

**Affiliations:** 1 Laboratory for Ground Stone Tools Research, Zinman Institute of Archaeology, University of Haifa, Haifa, Israel; 2 Institute of Archaeology, University of Wroclaw, Wroclaw, Poland; 3 The Use-Wear Analysis Laboratory, Zinman Institute of Archaeology, University of Haifa, Haifa, Israel; Ben-Gurion University of the Negev, ISRAEL

## Abstract

One of the most characteristic aspects of the Late Chalcolithic and Early Bronze Age periods in the southern Levant is the appearance of large assemblages of basalt vessels. These vessels, frequently meticulously made, appear sometimes a considerable distance from the raw material sources and are found mainly at habitation sites. While these and their prestigious value have been widely discussed in the past, their function is still obscure. In the current paper, we address their functionality through microscopic use-wear analysis. Emphasis was placed on basalt vessels with a distinct wear pattern–circumferential depressions, which appear along the perimeter of their interior bases. The documented traces were compared to results of an experimental study we conducted to characterize the effects of abrasion, grinding, and lubrication on basalt surfaces. The results of the comparative experimental study suggest that the circumferential depression was formed from a repetitive rotational activity using a narrow-ended tool. Further, it seems that two material types acted in combination as the circling device and processed material. One was hard and abrasive, such as stone, and the other was semi-resilient, such as wood or mineral powder. Water was likely used as a lubricant in the rotational process. While the actual function of the bowls bearing the circumferential depressions is not entirely clear, the use-wear analyses suggest that they may have been devices involved in craft industries, used for processing materials unrelated to food (minerals in particular). Whatever the exact function was, it clear that this use continued from the Chalcolithic through the Early Bronze Age, providing evidence for functional continuity between these two periods.

## 1. Introduction

The protohistoric southern Levant is characterized by notable economic and technological developments and important social changes [e.g., 1–3] that set the stage for early urbanism in the region. During the Late Chalcolithic (ca. 4,500–3,700 CalBC [4, 5]) and Early Bronze Age (ca. 3,700–2,500 CalBC [6]), there was an agro-pastoral economy based primarily on cereal and legume cultivation paired with animal herding, extending the Neolithic diet and economy to include secondary and horticultural products [e.g., 1, 7–15].

The social systems developed based on this economy reflect increasing social differentiation, craft specialization, and standardization [e.g., 3, 16–23]. By the Early Bronze Age, the mostly small-scale Chalcolithic settlements advanced in two trajectories. These include agglomerate often walled rural settlements and local urban centers with selective evidence for public structures and administrative control [e.g., 2, 24–28]. The latter occurred alongside increased public wealth and social stratification, which facilitated the emergence of the Early Bronze Age IB–III towns [e.g., 1–3, 9, 26, 29–35].

The transition between the Late Chalcolithic and the Early Bronze Age periods is marked by significant shifts in settlement distribution and material culture. The phenomenon has given rise to a debate over the continuity between the Late Chalcolithic and the Early Bronze Age [e.g., 3, 5, 36–41]. The Late Chalcolithic material culture, spectacular in its symbolic manifestations in art and mortuary practices [e.g., 42, 43] and marked by regional variability [e.g., 44–46], was replaced in the Early Bronze Age I with a simplified aniconic and utilitarian repertoire [e.g., 41, 47], observed in decoration, burial offerings, pottery and copper industries, and architectural conventions [e.g., 2, 17, 34, 37, 39, 48]. The phenomenon, initially understood as an expression of cultural regress, encouraged scholars to draw a sharp line between the cultural entities of these periods and look for the external motivation for such major changes [e.g., 34, 49–51]. While many gaps are yet to be filled, due to a growing scope of data in the last decade, the discussion has moved towards understanding the transition in a long-durée anthropological perspective. This approach views the Late Chalcolithic period as transitional itself, a culmination of the ‘neolithization’ process [52] characterized not only by its materialistic component–structurization into social groups, territorialization, a commodifying nature–but also by the cognitive layer of these changes, expressed through profoundly ritualized, totemic culture [41, 43, 53].

It has been suggested that the significant shifts in the material culture at the beginning of the Early Bronze Age reflect revaluation of socio-economic priorities imposed by an environmental or socio-economic pressure [e.g., 2, 41, 48, 54–56]. The urban economy of the Early Bronze Age evolves then from the materialistic achievements of the Chalcolithic, while restraining the symbolism inherent to the culture and turning it into an instrument of power [41, 57]. Indeed, the continuity between these periods is noticeable mainly when looking into pragmatic aspects of subsistence–expressed through economic and food procurement strategies and diet, but also aspects of tool making and portable material culture such as the composition of ceramic assemblages, the Canaanean blade, and the basalt vessel industries [e.g., 2, 3, 36, 37, 41, 58–61].

**Fig 1:**
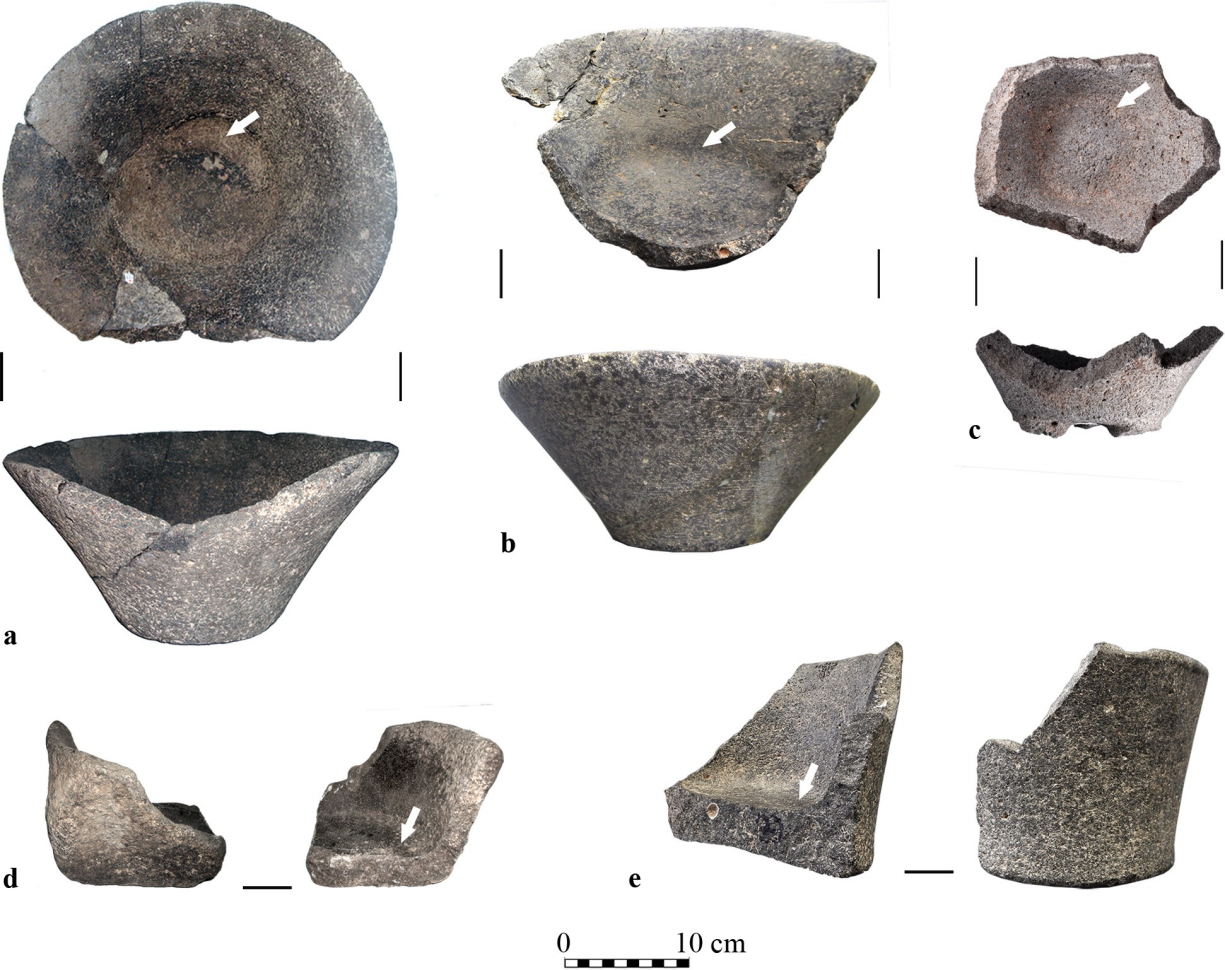
Examples of basalt vessels from the current study with a circumferential depression (marked by arrows). (a) V-shaped bowl from Giv’at HaOranim; (b) V-shaped bowl from Shiqmim; (c) Fragment of a fenestrated pedestal bowl from Namir Road; (d) Fragment of a four-handled bowl from Tel Bet Yerah; (e) Upright bowl from Modi’in.

**Fig 2:**
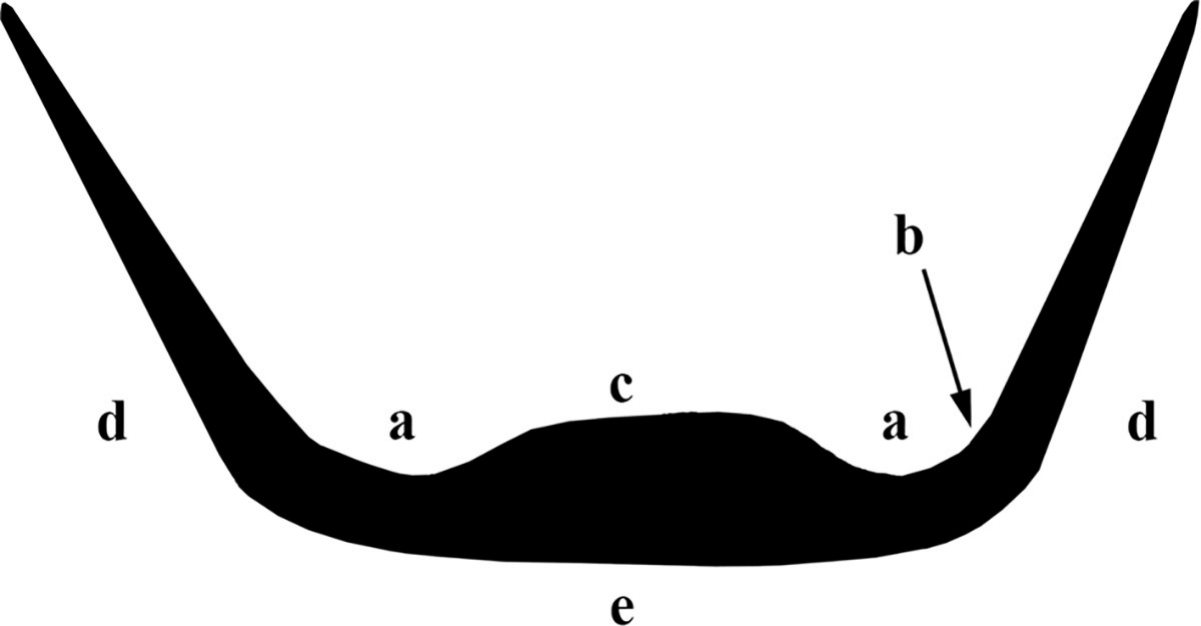
Schematic cross-section of a vessel with a circumferential depression. (a) Circumferential depression; (b) Exterior margin of the circumferential depression; (c) Central elevated base; (d) Exterior wall; (e) Exterior base.

The Late Chalcolithic and Early Bronze Age basalt vessel industries have attracted much attention in recent years [e.g., 23, 61–65]. While basalt vessels were commonly used in the southern Levant in earlier prehistory [see 66–68 and references therein], during the Late Chalcolithic period, a clear increase in the number of basalt vessels and their quality occurred [18, 69, 70]. Unlike many other strongly symbolic elements of the Late Chalcolithic material culture, the basalt vessel industry maintained an exceptional level of production consistency and stylistic refinement over the transition to the Early Bronze Age. Both Late Chalcolithic (Fig 1A–1C) and Early Bronze Age (Fig 1D and 1E) basalt vessels are thus characterized by a high-level of artisanship and finishing, with morphological and decorative standardization [20, 23, 61, 62]. The explanation for this remains unclear, but it most likely relates to the function (or functions) of these vessels and their social value. During the Late Chalcolithic, V-shaped bowls with either a flat base or a fenestrated stand were prevalent [e.g., 23, 70, 71], occasionally exhibiting incised triangular decoration on the interior rim or extensive external geometric decoration [18, 69]. They usually constitute the largest group of ground stone artefacts (between 48.7–62.5% for the published Late Chalcolithic assemblages included in the analysis). During the Early Bronze Age, the number of basalt vessels declined (down to between 8.9–22.7% based on the published Early Bronze Age assemblages included in the analysis), and more ordinary coarse vessels, mainly produced from limestone, became more common [e.g., 72–78]. This trend hints toward a recession in the basalt vessel industry. Accompanying this trend were changes in form and style. Some of the typical Late Chalcolithic forms, like the full or fenestrated pedestals, disappeared [however see Fig 2 in 79], and flat-based vessels with flaring walls and thick bases became dominant, alongside other distinct types such as the four-handled vessels [65 and references therein]. The vessels are also sporadically distinguished by their rare decoration formed in relief (also called a ‘necklace’ pattern, characterized by a ‘crested ridge band’ [70] or chain of knobs encircling the rim [e.g., 61, 62, 70]. Despite these changes, the specialized basalt vessel industry continued to form an important part of the Early Bronze Age I craft [e.g., 61, 62, 70].

All vessel types were found dispersed throughout modern day Israel and Jordan, although the four-handled vessels were by and large limited to the northern sites [65]. This wide distribution occurred despite the restriction of suitable basalt sources in the southern Levant to the Jezreel Valley, Galilee, Golan Heights, the Jordan Valley, and parts of northern Jordan and southern Syria [80–85].

The first stages of the vessel production likely used a hammerstone and chisel to roughly shape the external form of the vessel and hollow its interior, while the later stages used meticulous pecking and smoothing, which in many cases camouflaged the rougher production marks [23]. Pecking and stone-to-stone abrasion has been proven the most efficient and feasible techniques employed in a final stage of basalt tool manufacture [e.g., 86–90]. The production technology, especially when applied to produce vessels this large and symmetrical, with proportionally thin walls, great level of surface refinement, and sophisticated decoration, was incredibly time consuming and required a great deal of skills and experience. Therefore, it seems that these vessels were likely manufactured by specialists in workshops, located at or near basalt sources, and circulated through complex distribution networks [e.g., 3, 20, 23, 61–63, 70, 91–93].

The effort involved in raw material acquisition, high-risk production [22], and transportation probably ensured the vessels a high value [e.g., 20, 23, 61, 65, 70]. Their social significance and prestigious nature are occasionally reinforced by their incorporation into specific contexts, such as burial caves [e.g., 78, 94] and caches containing other prestigious objects [e.g., 61, 95–98]. Nonetheless, most both complete and fragmentary basalt vessels were found in settlement contexts and, including those deposited in burials, bear signs of use on their interior surfaces, such as striations, abrasion, polish, and rarely soot marks [23, 61, 94, 99, 100], suggesting a utilitarian purpose [e.g., 65].

Notably, a small fraction of the Late Chalcolithic and Early Bronze Age basalt vessel assemblages bear a specific feature: a circumferential channel-like depression (Fig 1, marked by arrows) that is located inside the bowls at the joint of the wall and base. This phenomenon is observed on up to only 14.3% of basalt vessel bases in a basalt vessel assemblage according to the selection of fully published sites (with the maximum number of four vessels with circumferential depressions identified in the assemblage of Tel Bet Yerah).

The current paper explores this unique feature through the application of use-wear analysis and experimental procedures. We present our observations and the results of the experiments we conducted and discuss several interpretive lanes regarding the mechanisms that may have formed this unique pattern. Finally, we address how this contributes to our understanding of the Late Chalcolithic and Early Bronze Age basalt vessels’ function.

## 2. Materials and methods

Use-wear analysis on basalt artifacts has advanced significantly during the last two decades, developing a standardized analytical procedure, terminology [e.g., 101–108], and wear reference collection [e.g., 88, 109–117]. These studies, which tend to focus on grinding implements, abraders, and grooved items [however see 118], yielded important results regarding the process of use-wear formation on basalt. Building on these studies, the current paper applies use-wear analysis to document and understand the phenomenon of the circumferential depressions inside the Late Chalcolithic and Early Bronze Age basalt vessels.

The depression is a shallow channel-like surface (Fig 2A), appearing along the perimeter of the vessel interior base where it adjoins the interior wall. In the most pronounced variation, the worn surface appears as a relatively deep circumferential depression with clear abrasion marks, parallel longitudinal striations, and highly reflective polish observable to the naked eye, especially on the bottom of the depression and its exterior margin (Fig 2B). In the least pronounced variation, the worn surface is particularly shallow. The depression is clearly distinguished from the central area of the interior base (Fig 2C), which is usually elevated in comparison to the perimeter.

**Fig 3:**
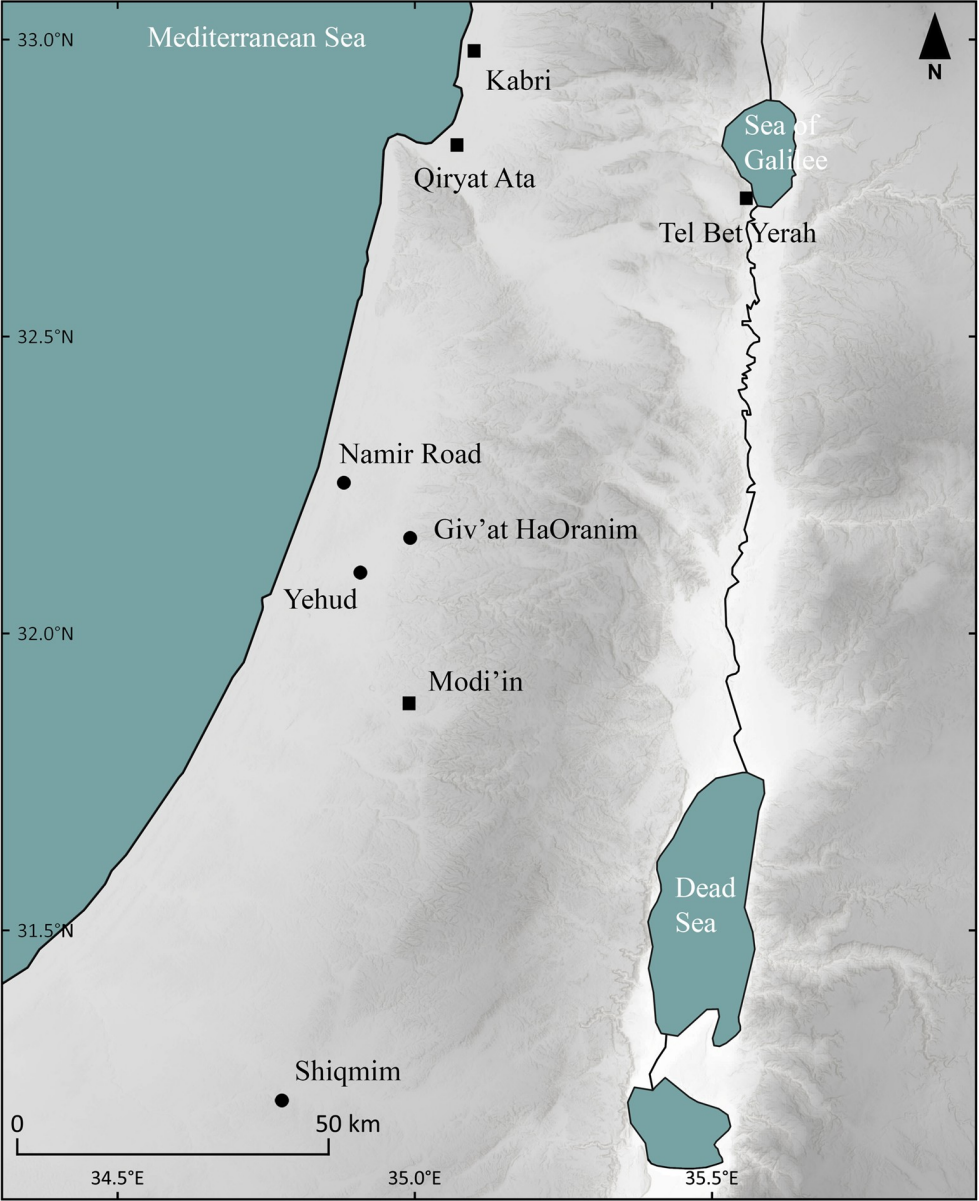
Location of the archaeological sites from which the examined vessels with circumferential depressions were studied. Late Chalcolithic settlements are marked with a circle and Early Bronze Age I settlements are marked with a square.

For the current study, we selected 15 vessels bearing a distinct circumferential depression, which originated from eight Late Chalcolithic and seven Early Bronze Age sites in Israel (Fig 3). These vessels originated from the recent excavations of the Israel Antiquities Authority or its storage facilities located in Beth Shemesh. No permits were required for the study, which complied with all relevant regulations.

The vessels were photographed, and their metric attributes were documented. The morphometric attributes of the circumferential depressions were measured using a digital caliper with a precision of 0.01 cm to calculate the average (using the minimum and maximum measures) depth and width. Further, five base fragments exhibiting pronounced circumferential depressions (three dated Late Chalcolithic and two to the Early Bronze Age I) were selected for comprehensive use-wear analysis. Of these, three specimens represent flat-based V-shaped bowls from the Late Chalcolithic sites of Yehud (YH9 [119], YH42 [120]) and Giv’at HaOranim (GHO1 [121]), and two fragments represent an upright/flaring bowl with a narrow base from Early Bronze Age I contexts of Tel Bet Yerah (TBY37 [77]) and a four-handled bowl from Kabri (KB; Israel Antiquities Authority collection) dated to the Early Bronze Age I. All the sampled vessels were produced of fine-grained compact basalt with olivine inclusions tightly embedded in the groundmass and minor-to-no natural porosity.

We applied the methodological framework of use-wear analyses, in which the characteristics of wear were defined using a list of attributes and then replicated through experimentation to infer on the mechanisms of their formation. We followed the protocols and attributes outlined by Adams [103, 104] for the low-power analysis and Dubreuil and Savage [88, 108] for the high-power analysis, with minor adjustments in terminology (Table 1).

**Table 1:** Attributes used to characterize the wear observed on the archaeological vessels and the experimental pieces [based on 88, 103, 104, 108].

**1**	**Basalt structure**		
	**Groundmass**	Fine-grained, almost homogenous, comprising isolated phenocrysts	
	**Phenocryst**	A large crystal embedded within the groundmass	
	**Olivine**	Green relatively transparent mineral, which usually appears as a large phenocryst in the basalt groundmass	
	**Pyroxene**	A group of black minerals common in igneous rocks	
**2**	**Microtopography: Low-power observations**	Morphology of the stone’s surface in cross-section	
	**Flat**	Topography profile exhibiting little elevation variation	**LEVEL 1**
	**Sinuous**	Topography profile with rounded and gradual transitions between low and high areas	
	**Uneven**	Topography profile with abrupt, rapid, and inconsistent transitions between low and high areas	
	**Regular**	Topography profile with minimal variation in the elevation of adjacent grains	**LEVEL 2**
	**Irregular**	Topography profile with pronounced variation in the elevation of adjacent grains	
	**Peak**	A high point of the microtopography	**TOPOGRAPHIC FEATURES**
	**Plateau**	A flattened peak	
	**Ridge**	An aggregation of peaks	
	**Pit**	Low points in the microtopography	
	**Recess**	An aggregation of pits	
**3**	**Wear features: Low- and high-power observations**	Alterations in the morphology of the stone’s surface or individual grains	
	**Leveling**	A uniform flat facet in relation to a surface or individual grains; the wear increases the dimension of high topography	
	**Darkening**	A darker area of the stone surface formed by exposure of fresh crystal grains through abrasion especially characteristic to flattened surfaces that create an effect of reduced scattering of the light off the stone surface	
	**Rounding**	Eliminated sharp edges and natural facets; in the description of an individual grain or transitions between high and low surface topography	
	**Fracture**	Fragmentation or cracks of the surface or individual grains. May occur as conchoidal or step fractures	
	**Comet-shaped pit**	A pit with a single worn side resulting from grains dragged in a single direction, indicating unidirectional abrasion	
	**Polish**	Visible alteration to the natural surface that increases its reflectiveness through leveling the microtopography and tribochemical reactions between working materials	
	**Pecking**	Conglomerates of steep shallow pits; the wear increases the dimension of low topography	
	**Striation**	An isolated, intermittent, or continuous track on the basalt surface created by grain dragged along the surface. May occur (regarding density) separated, close, or connected and (regarding spatial distribution) loose, covering, or concentrated	
**4**	**Micropolish: High-power observations**	Changes to the surface of a stone, which alter the reflectivity and microtopography of the crystal grains; the characteristics of micropolish are observable in high magnification	
	**Thickness**	The level of micropolish accumulation on the stone surface characterized relatively as thin or thick	
	**Brightness**	The ability of the micropolish to reflect light in comparison to the unmodified stone surface–dull or bright	
	**Opacity**	The visibility of crystals through the micropolish–opaque, translucent, or transparent	
	**Texture**	The asperity of micropolish associated with the thickness of the micropolish: rough, with asperities of the underlying surface visible in micropolish, fluid, with smoothed asperities of the underlying surface retaining the topography, and smooth, camouflaging the surface asperities	
	**Morphology**	Contour of the micropolish in cross-section–flat, sinuous, or irregular	
	**Distribution**	The extent and connectivity of micropolish–isolated, reticular, or covering	
	**Orientation**	When micropolish develops in a manner that reflects the direction of the actions that formed it	

The analyses were conducted at the Laboratory for Ground Stone Tools Research and at the Use-Wear Analysis Laboratory, both at the Zinman Institute of Archaeology, University of Haifa. An integrated observational approach, combining low- and high-power observations, was employed. For the low-power observations (at magnifications of 10–50x), a Zeiss DISCOVERY V8 stereomicroscope was used with the aid of a fiber optic light to provide a side-light source when presenting topography. High-power observations (at magnifications of 50–500x) were conducted using a Zeiss Scope.81 metallographic microscope. Photographs were taken using Z-stacking with a range of 10 to 30 shots that were merged into a full depth images, depending on the topography of the surface.

### 2.1 The analysis procedure

The analysis began with a thorough microscopic examination of the vessel surfaces to detect possible residue. As no residues were detected, the selected archaeological artifacts were washed in an ultrasonic tank filled with room-temperature water for 15 minutes to remove soil and crust (natural calcite deposits on the basalt surface). When dried, the microscopic observations used first low and then high-power observations. The unmodified breaks of the sampled archaeological pieces were observed first to assess post-depositional alternations. Following this, observations were applied to the circumferential depressions, the elevated central area of the bases, the exterior surface of the bases, and the preserved exterior wall of each of the five vessels analyzed. The interior walls of the sampled vessels are not preserved so were not analyzed. Various spots exhibiting distinct features were photographed for each vessel to show the variations in traces, and these features were defined using the list of attributes presented in Table 1.

### 2.2 The experimental procedure

The experimental program designed for the current study was aimed particularly at replicating the mechanism for the formation of the traces observed on the archaeological specimens, associated with the formation of the circumferential depressions (surface abrasion, parallel striations, and polish). The preliminary observations conducted on the archaeological samples showed that the wear within the circumferential depressions is indicative of a friction between two parallel surfaces; therefore, all the experiments were conducted using grinding. We decided to test basalt against (1) high-asperity rock types to study the abrasion of basalt groundmass, (2) various types of plants to check how and to what extent grinding organic products of various hardness alters the compact basalt surface, and (3) lubricants to understand their influence on the abrasion mechanism and the development of micropolish. The basalt used for the experiment was collected from the Golan Heights. The region constitutes the westernmost part of the Transjordanian Harrat Ash Shaam volcanic province, which is rich in high-quality basalt. The area likely served as the raw material source for production of many of the Late Chalcolithic and Early Bronze Age basalt vessels [64, 81, 93, 122].

Since the basic assumption on which the first stage of the experiment was designed is that use-wear on stone vessels is formed on top of production traces, we overlapped the various wear procedures through sequential operations on the same stones. The experiments therefore included two stages. The first was an abrasion experiment, which was aimed to produce a smooth surface to simulate the vessels’ production wear on which the use-wear is assumed to develop. The second was a grinding experiment where the abraded stones with the smoothed surfaces were used as the lower grinding stones, and various materials were grinded on top of them. This stage simulated the formation of use-wear on top of production wear. In this experiment, a subset of experiments was included to test the effects of lubrication on the interaction between the upper and lower stone and the use-wear. Lubrication is hypothesized to enhance the rotational action, which was assumed to be involved in the formation of gloss and the depression on the archaeological tools (Table 2).

**Table 2:** The experimental program showing the sequence of operations and the materials used for abrasion, grinding, and lubrication.

**Experimental stone**	**Stage 1**		**→**	**Stage 2**	
	**Abrasion experiment**			**Grinding experiment**	
	**Material**	**Duration (min)**		**Material**	**Duration (min)**
**1**	Aeolianite	80		Oat	120
**2**	Aeolianite with sand	50		Rosemary	150
**3**	Beachrock	3		Flax seeds	120
				**Lubricant experiment**	
**4**	Aeolianite with sand	20		Sand with water	60
**5**	Beachrock with sand	25		Limestone with water	120
**6**	Beachrock	20		Sand with oil	60

In the first stage of the experiments, two highly abrasive and locally accessible stones were selected to create the smooth surface: well-cemented beachrock and aeolianite sandstone (also called *kurkar*). Both are composed of gravel, shells, and quartz sand, which make them particularly efficient in smoothing hard rocks. Quartz sand was also used as an additive, and altogether these materials were tested in various combinations. The selection of contact materials was driven by the microscopic observations of the archaeological vessels and their surface characteristics, cross-referenced with ethnographic and experimental studies on basalt tools manufacture and use. The experiments were carried out until a smooth surface was formed on the basalt pieces.

**Fig 4:**
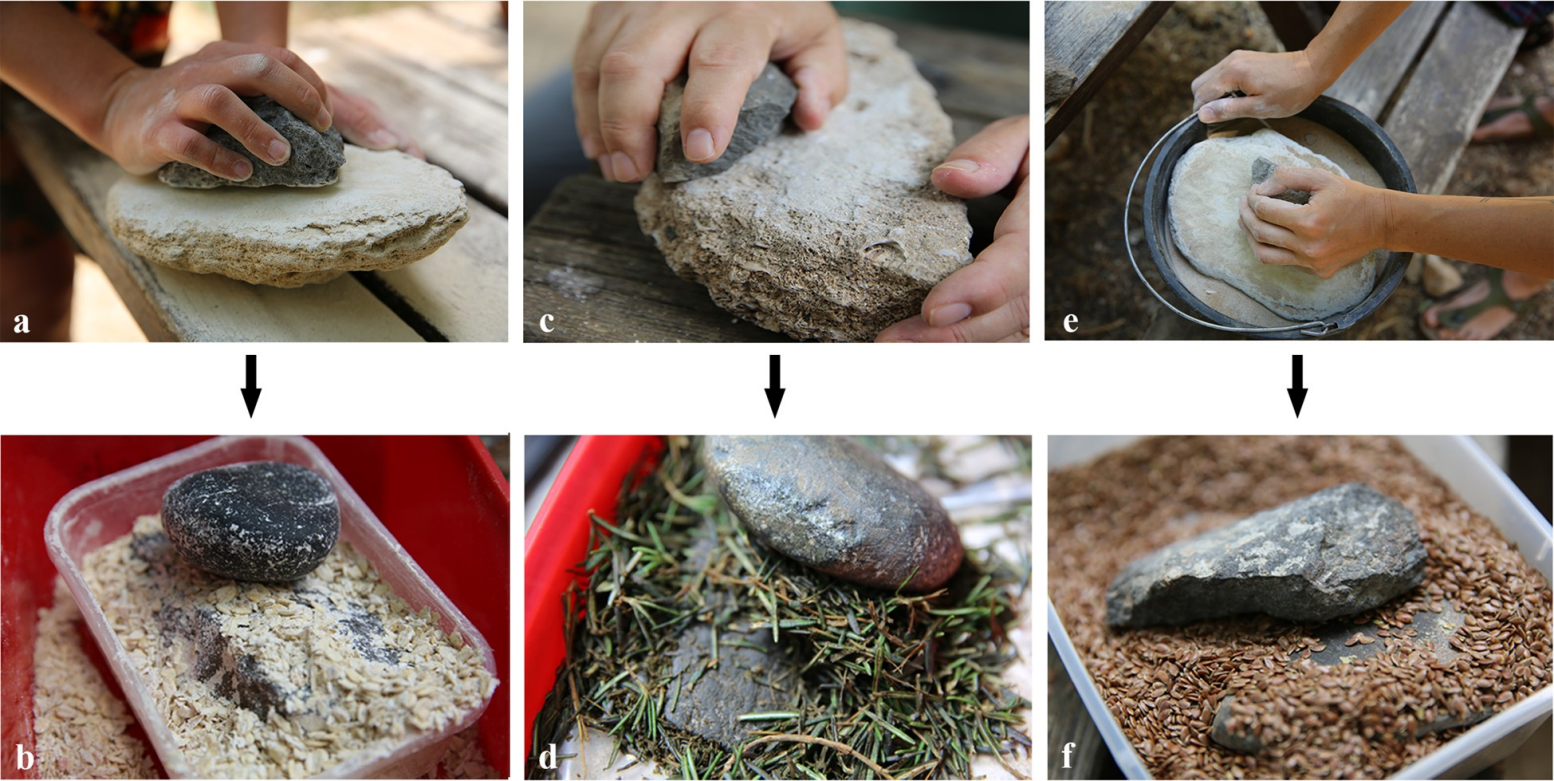
Experimental program. (a) Abrading basalt against aeolianite; (b) Grinding oat flakes on the surface prepared by abrading the aeolianite; (c) Abrading basalt against beachrock; (d) Grinding rosemary leaves and stems on the surface prepared by abrading against beachrock; (e) Abrading basalt against aeolianite with sand; (f) Grinding flax seeds on the surface prepared by abrading against aeolianite with sand (see also Table 2).

The smoothed surfaces were documented and then used as the lower stones in the second stage of the experiments where three plant materials were ground (Fig 4). The plants selected represent different levels of pliability and moisture; fresh rosemary stems represent a relatively oily plant and a fresh wood, dry oat flakes represent a soft and dry flour-generating plant, and dry flax seeds represent lipid-rich seeds with a relatively hard shell (Table 2). In this stage, for the grinding of the plants, raw pieces of compact basalt slabs served as upper grinding stones. These experiments were carried out until a handful of the processed end-product was acquired.

Finally, the lubricant experiments involved abrading basalt against sand and water, sand and oil, and wet limestone. Sand was used since it provides the abrasiveness and surface fatigue factors, which according to our preceding abrasion experiment (Table 2) was successful in enhancing smoothing, with minor influence on the reflectiveness. The experiment with limestone and water aimed to create a shiny surface using both a lubricant and a soft but compact stone.

The duration of the experiments was decided based on our observations, up to a point when wear was observed with the naked eye. Before conducting the microscopic analysis, all experimental stones were soaked in soap water for at least five minutes (up to two hours in the case of stones involved in the experiment with oily materials) and cleaned for 15 minutes in an ultrasonic tank filled with room-temperature water. The traces were documented and analyzed following the same protocol established for the archaeological vessels. The final stage of analysis compared the wear patterns on the experimental pieces with the archaeological vessels. The results of microscopic observations were also cross-referenced with past experiments targeting use-wear on basalt tools [e.g., 88, 101–104, 108–110, 115].

## 3. Results

### 3.1 The experimental pieces

**Fig 5:**
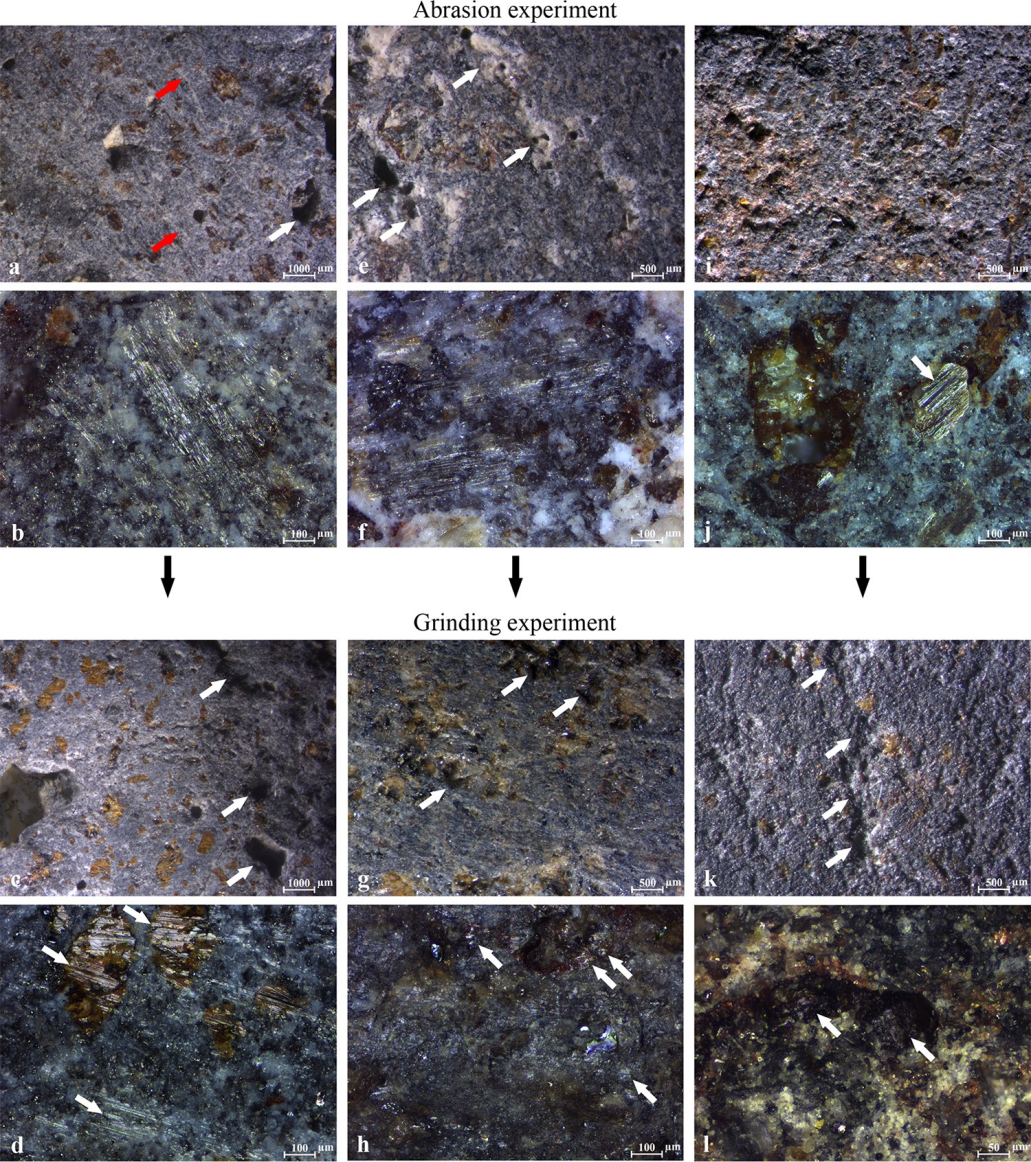
Macro- and micro-scale use-wear observed on the experimental basalts. (a, b) Traces by abrading with aeolianite: (a) Flat leveled surface, isolated amorphous pits with sharp edges marked by an arrow, and striations marked by red arrows (10x); (b) A polished surface with a patch of connected intermittent striations (100x); (c, d) Traces by grinding oat on the surface abraded with aeolianite: (c) Sinuous regular surface and pits with rounded edges marked by arrows (10x); (d) A polished surface associated with flat bright striated patches of polish produced by the contact with the upper stone, marked by arrows (100x); (e, f) Traces by abrading with aeolianite and sand: (e) Sinuous regular surface and diffuse pits with rounded edges and bottoms marked by arrows (20x); (f) A polished surface with patches of connected intermittent striations (100x); (g, h) Traces by grinding rosemary on the surface abraded with aeolianite and sand: (g) Flat regular surface and pits with relatively sharp edges marked by arrows (20x); (h) Translucent irregular covering polish, developing a reticular pattern on protruding surfaces, marked by arrows (100x); (i, j) Traces by abrading with beachrock: (i) Flat rough irregular surface and amorphous pits with irregular bottoms (20x); (j) A patch of flat striated polish on top of the olivine, marked by an arrow (100x); (k, l) Traces by grinding flax seeds on the surface prepared with beachrock: (k) Flat irregular surface, protruding topography moderately rounded, and diffuse recesses with rough edges and bottoms marked by arrows (20x); (l) Thin, rough, dull, transparent polish covering the surface and patches of developing reticular pattern marked by arrows (100x) (see also Tables 3 and 4).

The experiments produced wear linked to the hardness and asperity of the contact materials and lubrication (Fig 5). The use of abrasive rocks, with or without the addition of sand, for the preparation of the experimental lower stones produced diagnostic wear. In each case we obtained working surfaces that appeared well-flattened with a relatively dull reflection visible to the naked eye. Depending on both the applied method (beachrock vs aeolianite vs aeolianite or beachrock with the addition of sand) and the original topography of basalt rock, this task took 3–120 minutes. The specific characteristics of the wear for each stone are listed in Table 3.

**Table 3:** Description of traces produced in the first abrasion experiment (detailed in Table 2).

Product	Profile		Topography		Striations	Grain alteration	Micropolish	Fig
	Level 1	Level 2	High	Low				
**Aeolianite**	Flat	Regular	Large plateaus	Amorphous pits with sharp edges and unmodified bottom	Parallel, intermittent, loose but connected, appearing in patches	Leveled	Isolated, flat, translucent, bright, and thin, appearing in patches with parallel striations	5a and 5b
**Aeolianite with sand**	Sinuous	Regular	Large plateaus with rounded edges	Diffuse pits with rounded edges and bottoms (in particular the shallow ones)	Parallel, intermittent, loose but connected, appearing in patches	Rounded	Isolated, flat, translucent, dull, and thin, appearing in patches with parallel striations	5e and 5f
**Beachrock**	Flat	Irregular	Large plateaus with irregular surface	Amorphous pits with irregular bottoms	Parallel, intermittent, loose but connected, appearing in patches on phenocrysts	Fractured	Isolated, flat, translucent, bright, and thin, appearing in patches with parallel striations	5i and 5j

**Table 4:** Description of traces produced in the second grinding and lubricant experiment.

Product	Profile		Topography		Striations	Grain alteration	Micropolish	Figs
	Level 1	Level 2	High	Low				
**GRINDING EXPERIMENT**								
**Oat flakes**	Sinuous	Regular	Large plateaus with rounded edges	Maintained original shape, rounding	Intermittent loose striations	Rounded	Thin, rough, dull in reflectivity, transparent, covering	5c and 5d
**Rosemary**	Flat	Regular	Large plateaus	Maintained original shape, rounding	Loose, continuous striations on the plateaus	Rounded	Translucent, irregular, covering; fine reticulation at protruding surfaces	5g and 5h
**Flax seeds**	Flat	Irregular	Large plateaus	Shallow diffuse recesses, rough surface	No linear features	Rounded, grain extraction	Thin, sinuous, dull in reflectivity, transparent, covering; fine reticulation at protruding surfaces	5k and 5l
**LUBRICANT EXPERIMENT**								
**Sand with water**	Flat	Regular	Plateaus with rounded edges	Isolated pits with rounded edges	Superficial, parallel striations	Rounded	Patches of thick, bright, opaque, flat, reticular polish	6a
**Sand with oil**	Flat	Regular	Plateaus with rounded edges	Isolated pits with rounded edges	Superficial parallel striations	Rounded	Patches of thick, dull, opaque, flat, reticular polish	6b
**Limestone with water**	Sinuous	Regular	Small plateaus and rounded peaks	Diffuse, irregular recesses with rounded sides	Parallel, continuous, covering striations on protruding surfaces	Leveled	Patches of thick, bright, opaque, flat, reticular polish; patches of rough, striated polish on protruding surfaces	6c

Abrasion against aeolianite generally produced traces indicative for contact with a hard material (Table 3), including flat plateaus, pits with sharp edges, and leveled surfaces. The abrasion of aeolianite with the addition of sand caused a greater reduction of the surface matrix but a more rounded topography as sand enabled a more fluid motion against the aeolianite and caused the collapse of sharp edges and grain removal. Beachrock caused a more significant reduction of the matrix, creating a rough and irregular topography.

All the abraded pieces were used in the subsequent experiments involving grinding plants and lubrication, and the details of the wear are shown in Table 4. Processing plant materials removed traces produced during the abrasion experiments. Grinding oat flakes generated traces typical for highly pliable products; the surface that was originally flat became sinuous, with pits with rounded edges. In addition, patches of a flat, bright, and striated polish formed on protruding surfaces and phenocrysts (Fig 5D, marked by arrows), resulting from occasional direct contact with the upper stone. Grinding rosemary changed the sinuous profile of the surface into a flat one, with pits showing relatively abrupt edges and polish typical of woody plants (Fig 5H). Grinding flax rounded and smoothed the previously rough surface, and elevation variation caused by the beachrock was reduced. A polish with the characteristics typical for beachrock abrasion covered the surface, with isolated patches of duller and sinuous polish from the flax grinding (Fig 5L).

**Fig 6:**
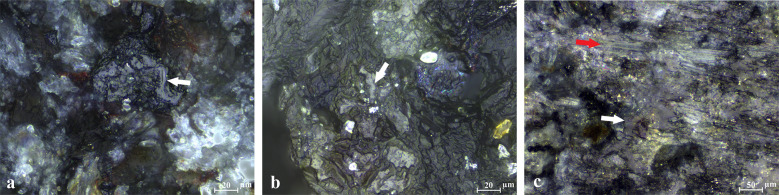
Micropolish produced in the lubricant experiment. (a) Traces from sand and water, showing a patch of bright polish in reticulation marked by an arrow (500x); (b) Sand and oil traces, showing a patch of moderately reflective polish marked by an arrow (500x); (c) Limestone and water traces, showing a combination of bright, rounded, and reticular polish formed from contact with the lubricant, marked by a white arrow, and flat striated opaque polish caused by contact with the hard limestone, marked by a red arrow (200x) (see Table 4).

Grinding with sand mixed with a lubricant (Fig 6A and 6B) allowed a more fluid motion and generated particularly well-smoothed surfaces (to an even higher level than with dry sand), clearly enhancing the abrasion. Traces on the macro and micro-scale are more or less the same for the experiments with water or oil, with minor differences visible only under a very high magnification (500x). The polish produced by sand and oil (Fig 6B) is less reflective than polish produced by sand and water (Fig 6A), suggesting that water is the component enhancing reflectivity. In the case of the limestone and water (Fig 6C), the polish differs and is slightly less reflective than the polish formed by water and sand, exhibiting a combination of flat and striated patches (Fig 6C, shown by the red arrow) and smooth and reflective sections (Fig 6C, shown by the white arrow).

To summarize, the experiments provided the wear reference collection that allows to proceed with the analysis of the archaeological artifacts. The most relevant conclusions include:

1. The use of sand generates a leveled topography with rounded transitions between plateaus and pits and less pronounced linear features (striations), while abrading compact abrasive rocks without the intermediate sand generates large plateaus with sharp edges and (in particular beachrock) is more likely to cause grain fracturing and irregular topography;
2. Beachrock is more efficient in matrix reduction and abrading basalt surface than aeolianite, while the presence of sand enhances the abrading effectiveness (in particular of aeolianite) and surface leveling;
3. Grinding plant materials removes or reduces the traces generated by the rock abrasion in the previous experimental phase and enhances the development of polish on different elevations of the microtopography;
4. Flattening the treated surfaces in the first experimental phase allows higher rate of polish development during the subsequent procedure;
5. The traces observed at the final stage may result from the contact with the upper stone as well, therefore exhibiting a combination of wear formed from different origins. This was observed in particular when the intermediate product is very pliable and fine (like oat flakes, water, or water with limestone powder);
6. Grinding rosemary resulted, among others, in wear features indicative for basalt-to-wood interaction–in particular fine reticulation of opaque and bright polish on the most exposed protruding surfaces;
7. Grinding relatively resilient oil-rich organic products (rosemary and flax seeds) enhances the development of reticular polish on protruding areas of the basalt topography;
8. The presence of lubricant–both water and oil–enhances reticulation of the micropolish. Water additionally enhances its reflectivity;
9. The combination of abrasive mineral (sand or limestone powder) with lubricant both reinforces the abrasion of basalt surface and facilitates the development of reticular polish.

### 3.2 Archaeological samples

#### 3.2.1 General observations

**Fig 7:**
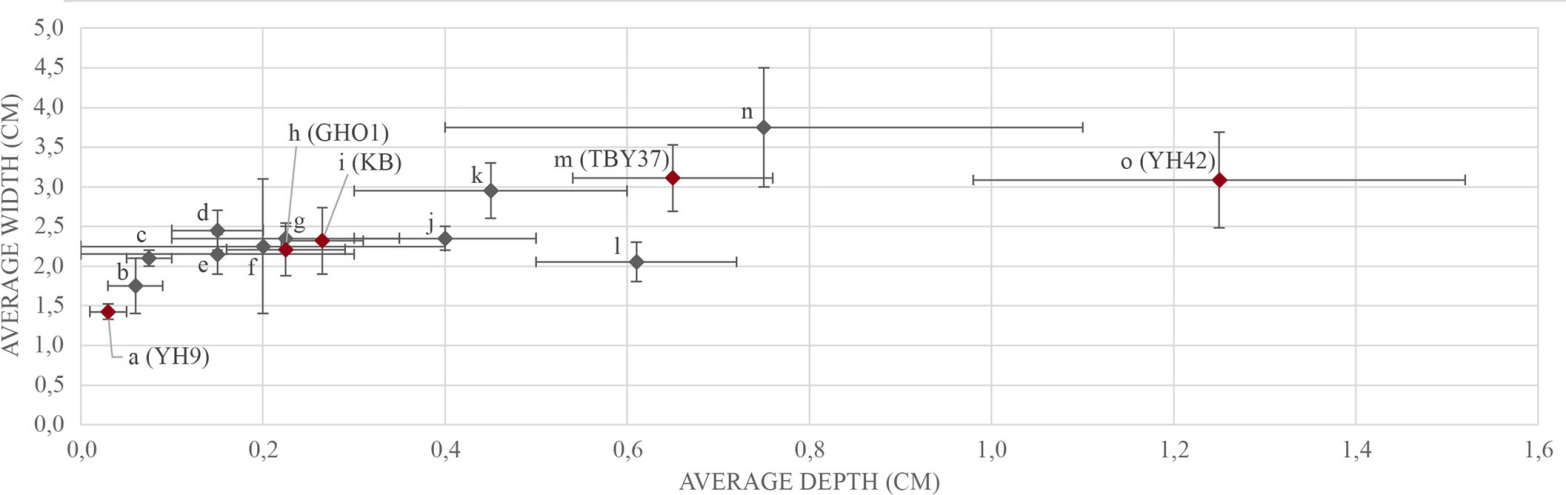
Scatterplot displaying the width and depth of the circumferential depressions. (a) Yehud 9, license B-327/2008 (YH9), loc. 31, reg. no. 31; (b) Qiryat Ata 32, loc 3019, reg. no. 4071; (c) Shiqmim, reg no. 1159.07; (d) Tel Bet Yerah 36, loc. UN 670, reg. no. 176; (e) Tel Bet Yerah 41, loc. UN 029, reg no. 358–50; (f) Giv’at HaOranim, loc 1178, reg. no. 2341; (g) Modi’in 130, loc. 3040, reg. no. 2154; (h) Giv’at HaOranim 1 (GHO1), loc. 4136, reg. no. 3600; (i) Kabri (KB), context unknown; (j) Giv’at HaOranim, loc. 1288, reg. no. 3211; (k) Tel Bet Yerah 42, loc. GB reg. no. 50–7473; (l) Namir Road 46, loc. 121/422 reg. no. 2116; (m) Tel Bet Yerah 37 (TBY37), loc. GB, reg. no. 50–3229; (n) Giv’at HaOranim 2, loc. 1521, reg. no. 4402; (o) Yehud 42, license A-8111/2017 (YH42), loc. 305, reg. no. 2162.

Based on the 15 examined vessels (including the five vessel fragments selected for use-wear analysis), the average depth of the circumferential depressions is 0.37 cm (standard deviation 0.33 cm), and the average width is 2.42 cm (standard deviation 0.59 cm). The average dimensions and the range of variation for each artifact are presented in Fig 7. The majority of the depressions oscillate between 1.40 cm and 3.50 cm in width and are up to 0.80 cm deep, with two particularly large exceptions, which are on average 3.75 cm wide (Fig 7N) and 1.25 cm deep (Fig 7O).

The use-wear analysis, applied to the five selected vessel fragments (YH9, YH42, GHO1, TBY37, and KB), showed that the break areas of each of the vessels present little to no alteration, retaining the original structure of the raw material. It was therefore established that post-depositional processes did not significantly affect the appearance of production- or use-wear.

**Fig 8:**
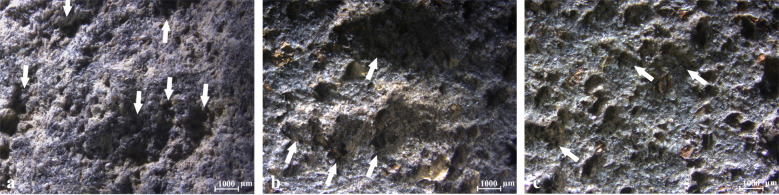
Wear typical to the vessels’ exterior. (a) Exterior wall of the Early Bronze Age I vessel fragment from Tel Bet Yerah showing pecking marked with arrows (10x); (b) Exterior wall of the Late Chalcolithic vessel fragment from Yehud showing abraded pecking marks marked with arrows (10x); (c) Exterior base of the same bowl from Yehud exhibiting extremely abraded pecking marks marked with arrows (10x).

The comparison of the surfaces of the exterior wall and base and the interior central elevated base to the circumferential depression revealed that the exterior walls and bases exhibit similar wear: abraded surfaces and pecking marks (Fig 8) that are less pronounced on the exterior base (Fig 8C) than the wall or the exterior wall-base joint (Fig 8A and 8B). Such wear is sometimes evident on the interior base as well, within the elevated center. The basalt topography in this area exhibits a more sinuous profile than within the circumferential depression, with pronounced and irregular pits. The pecking marks were largely abraded and leveled, likely during the final stages of vessel manufacture, aimed at forming clear smoothed surfaces [23, 88]. They could be further worn down during the use-life of the vessel or through holding the vessel exterior or resting it against a surface. The use of the vessel interior for processing or storing would contribute to abrasion of the pecking marks visible on the central elevated base; therefore, they are evident to various levels in different vessels and entirely missing in the circumferential depressions.

#### 3.2.2 The circumferential depression

The circumferential depression differs remarkably from the other surfaces of the vessels. It exhibits no evidence of pecking, and it is more heavily abraded, with a flatter topography, smoothed and heavily striated surfaces, developed polish, and occasionally surface darkening. The traces tend to intensify towards its bottom and the exterior margin (Fig 2B) that adjoins to the vessel wall.

The five vessels sampled were described and discussed separately, focusing on characterization of the traces within the circumferential depression (Table 5).

**Table 5:** Description of the traces observed within the circumferential depression of each of the five archaeological vessels analyzed for use-wear.

Vessel	Profile		Topography		Linear traces	Grain alteration	Micropolish	Figs
	Level 1	Level 2	High	Low				
**YH9 (LC)**	Sinuous	Irregular	Plateaus with rounded edges	Deep and shallow pits and diffuse recesses with rounded edges and irregular bottom	Connected, covering parallel striations	Rounded	Thin and translucent or thick and opaque, bright, rough, irregular, covering	9
**YH42 (LC)**	Flat	Regular	Large plateaus with rounded edges	Deep pits and diffuse recesses with rounded edges and irregular bottom	Macro-scale striations parallel, continuous, connected, and covering. Micro-scale striations are parallel and connected, distributed in loose patches on elevated surfaces	Rounded	Thick, opaque, bright, smooth, irregular, reticular, in patches on elevated surfaces	10
**GHO1 (LC)**	Flat	Regular	Large, flat plateaus, surface darkening	Deep and shallow isolated comet-shaped pits with sharp edges and irregular bottoms	Macro-scale striations continuous, connected, parallel, and covering. Micro-scale striations are parallel, connected, distributed in loose patches on elevated surfaces	Leveled	Thick, opaque, bright, smooth, irregular, in isolated patches on elevated surfaces	11
**TBY37 (EB)**	Flat	Regular	Large, flat plateaus, surface darkening	Deep but mostly shallow pits with sharp or irregular edges	Continuous, parallel, connected, covering macro-scale striations. Micro-scale striations distributed in loose patches on elevated surfaces	Leveled	Thick, bright, opaque, sinuous, reticular	12
**KB (EB)**	Flat	Regular	Large, flat plateaus, surface darkening	Deep and shallow, isolated, irregular pits with sharp edges and irregular bottoms	Continuous, parallel, connected, and covering macro-scale striations	Leveled	Thick, bright, opaque, sinuous, reticular	13

**Fig 9:**
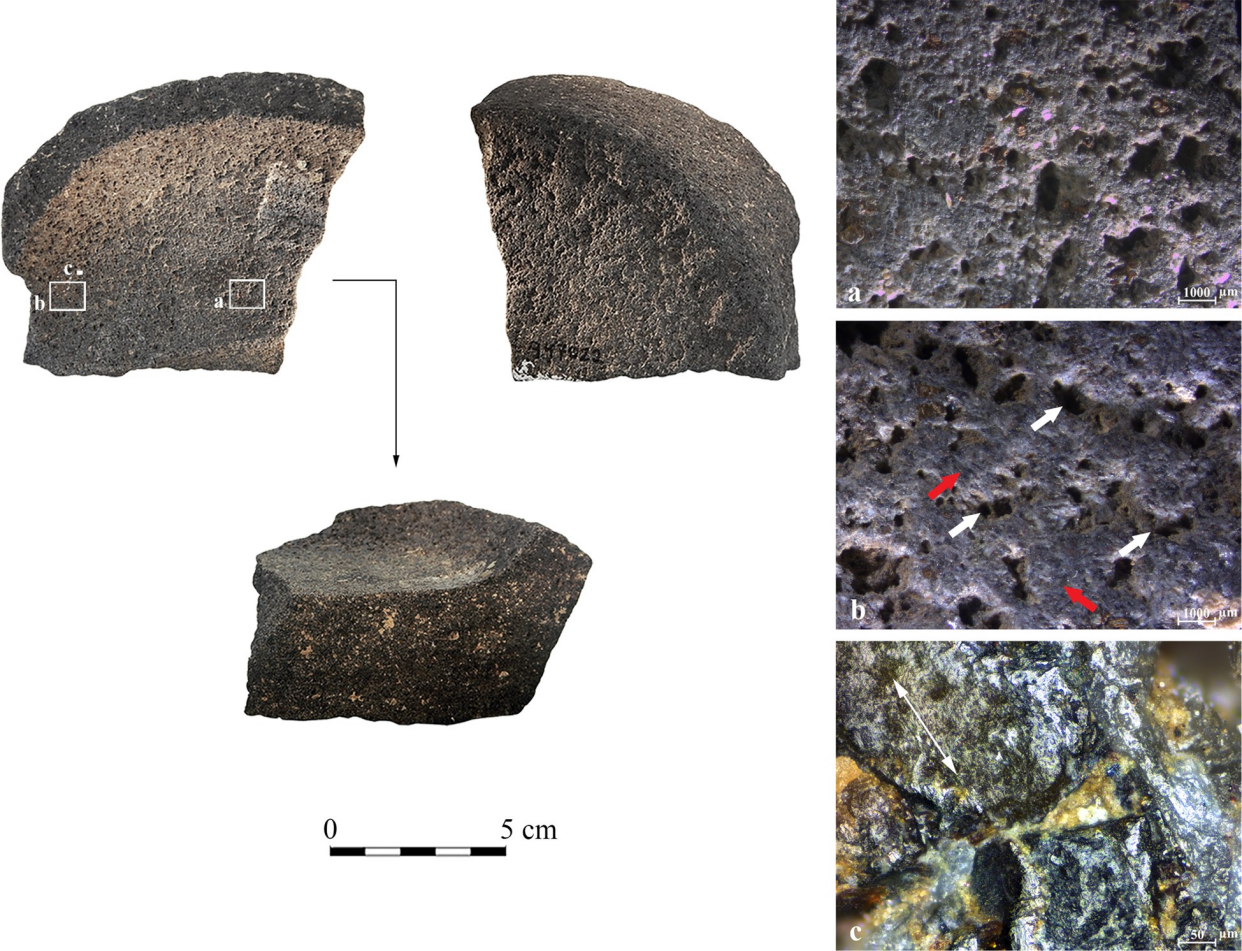
Archaeological vessel YH9 from the Late Chalcolithic site of Yehud. (a) Traces on central elevated base, displaying small plateaus and rounded pits (10x); (b) The bottom of the circumferential depression, exhibiting abraded and rounded plateaus (red arrows) and rounded pits (white arrows) (10x); (c) A polished surface within the circumferential depression with a connected pattern of continuous striations, their orientation marked by a double-headed arrow (200x).

**YH9** (Fig 9): the Late Chalcolithic base fragment shows a relatively superficial circumferential depression compared to the other vessels (Fig 7). The shiny appearance of the depression is notable and evident to the naked eye. On the macro-scale, traces in the depression are similar to those observed on the central elevated base (Fig 9A); however, in the depression they are more pronounced (Fig 9B; Table 5). The polish is spreading on wide surfaces (Fig 9C), developed to a higher degree on elevated areas and associated with fine striations. The striations appear throughout the surface of the depression, parallel to its main axis. Our experiments suggest that such a moderate change in surface topography combined with the spreading of the polish may result from contact with a medium-hard material.

**Fig 10:**
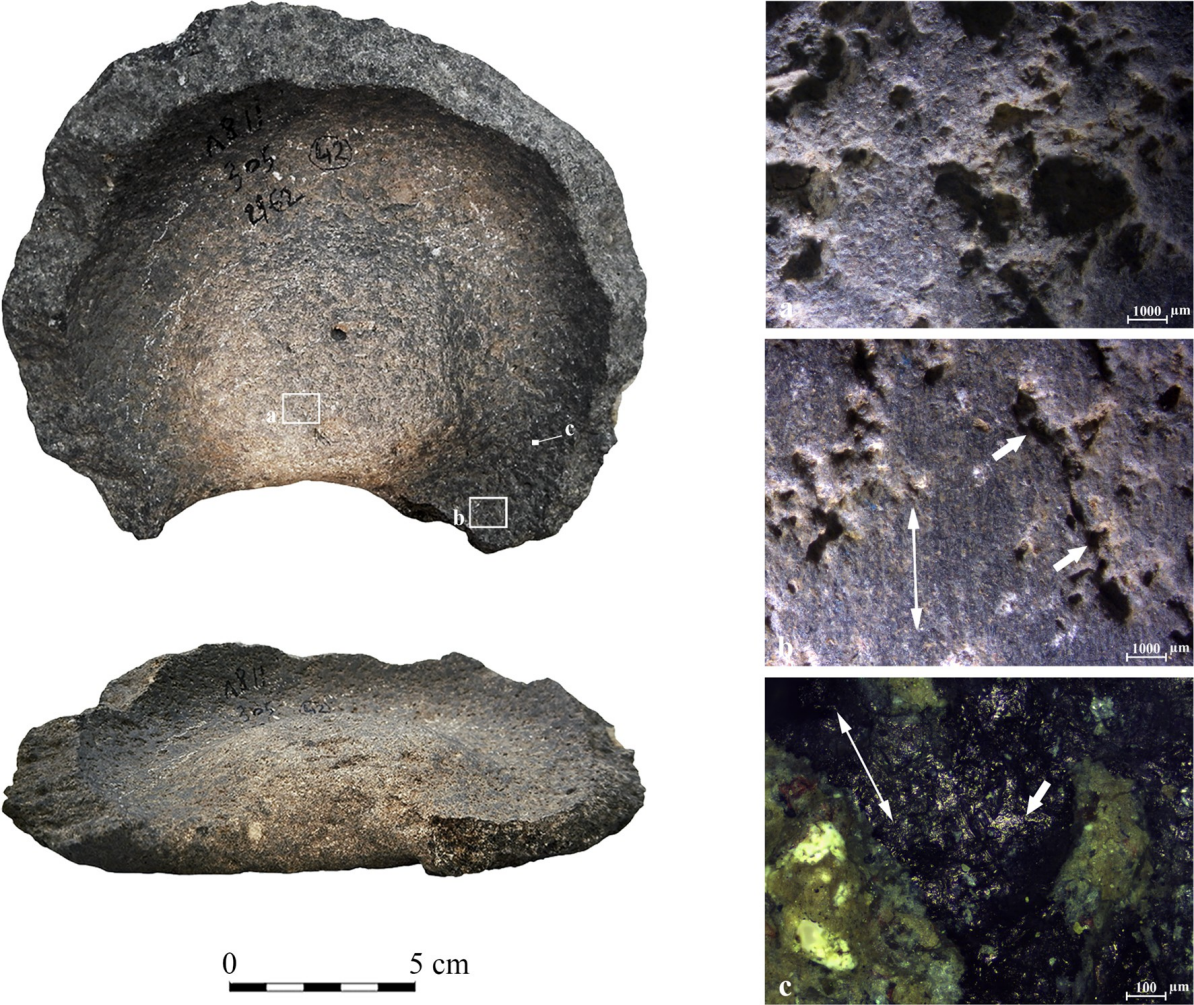
Archaeological vessel YH42 from the Late Chalcolithic site of Yehud. (1) Sinuous surface of the elevated center with small flattened peaks and rounded pits (10x); (2) Surface of the circumferential depression with large plateaus, diffuse rounded recesses marked by arrows, and continuous parallel covering striations, with their orientation marked by a double-headed arrow (10x); (3) Concentrations of developing reticular polish on the protruding surfaces within the circumferential depression, marked by arrows (the orientation of the macro-scale striations marked by a double-headed arrow) (100x).

**YH42** (Fig 10): the Late Chalcolithic base fragment is nearly complete with partially preserved walls. The circumferential depression is the deepest among the analyzed vessels examined for use-wear (Fig 7). Compared to the central elevated base (Fig 10A), the surface within the depression exhibits a marked change, with wide striated flat surfaces and reduced pits (Fig 10B; Table 5). The micropolish is generally developed to a relatively low degree, observed in patches on protruding topography (Fig 10C). Striations appear all over the circumferential depression, parallel to its axis. Based on our experiments, this combination implies that a relatively hard material was rotated along the depression, causing a massive reduction of the basalt groundmass. The prominent reduction compromised the formation of a well-developed polish, allowing its development only in isolated areas.

**Fig 11:**
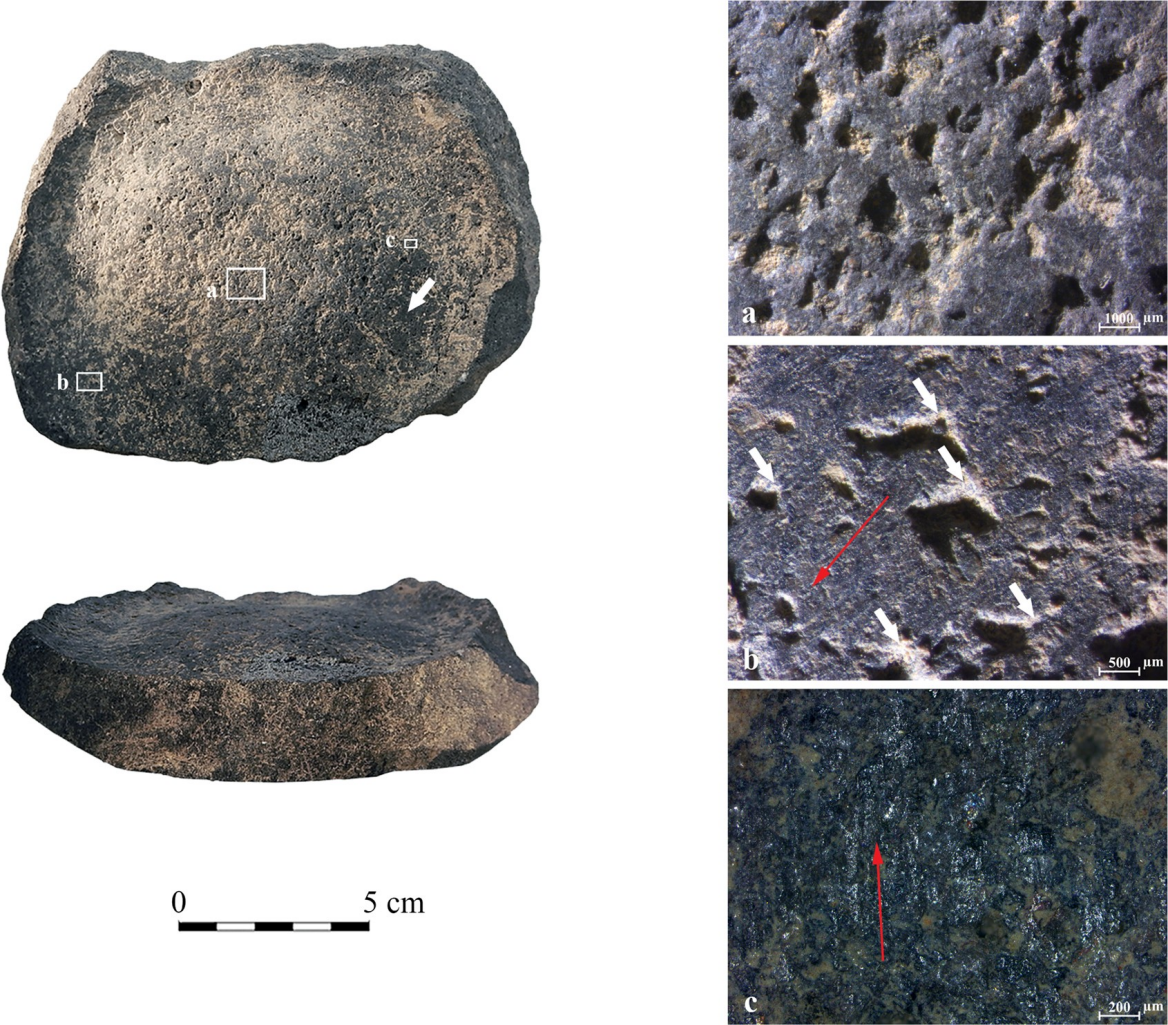
Archaeological vessel GHO1 from the Late Chalcolithic site of Giv’at HaOranim, with surface darkening marked by an arrow. (a) Sinuous surface of the elevated center with small flattened peaks and rounded pits (10x); (b) Flat striated surface within the circumferential depression with comet-shaped pits marked by white arrows. The direction of traces is marked by the red arrow (20x); (c) Thick opaque polish distributed in isolated patches on protruding surfaces. The patches show an orientation consistent with the striations, and their direction is marked by the red arrow (50x).

**GHO1** (Fig 11): the Late Chalcolithic complete vessel base preserved with minor parts of the vessel’s wall. The width and depth of the circumferential depression are typical when compared to the other examined vessels (Fig 7). Contrary to the central elevated base (Fig 11A), the circumferential depression exhibits massively worn flat surfaces with macro- and micro-scale parallel striations oriented along the depression (Table 5). Significantly, comet-shaped pits (Fig 11B) indicate a unidirectional motion of abrasion (Fig 11B). The direction of the comets corresponds with the direction of the striations (Fig 11C), and none of these appear on the central elevated base. The polish is developed along the protruding surfaces between the macro-scale striations, indicating contact with a hard material.

**Fig 12:**
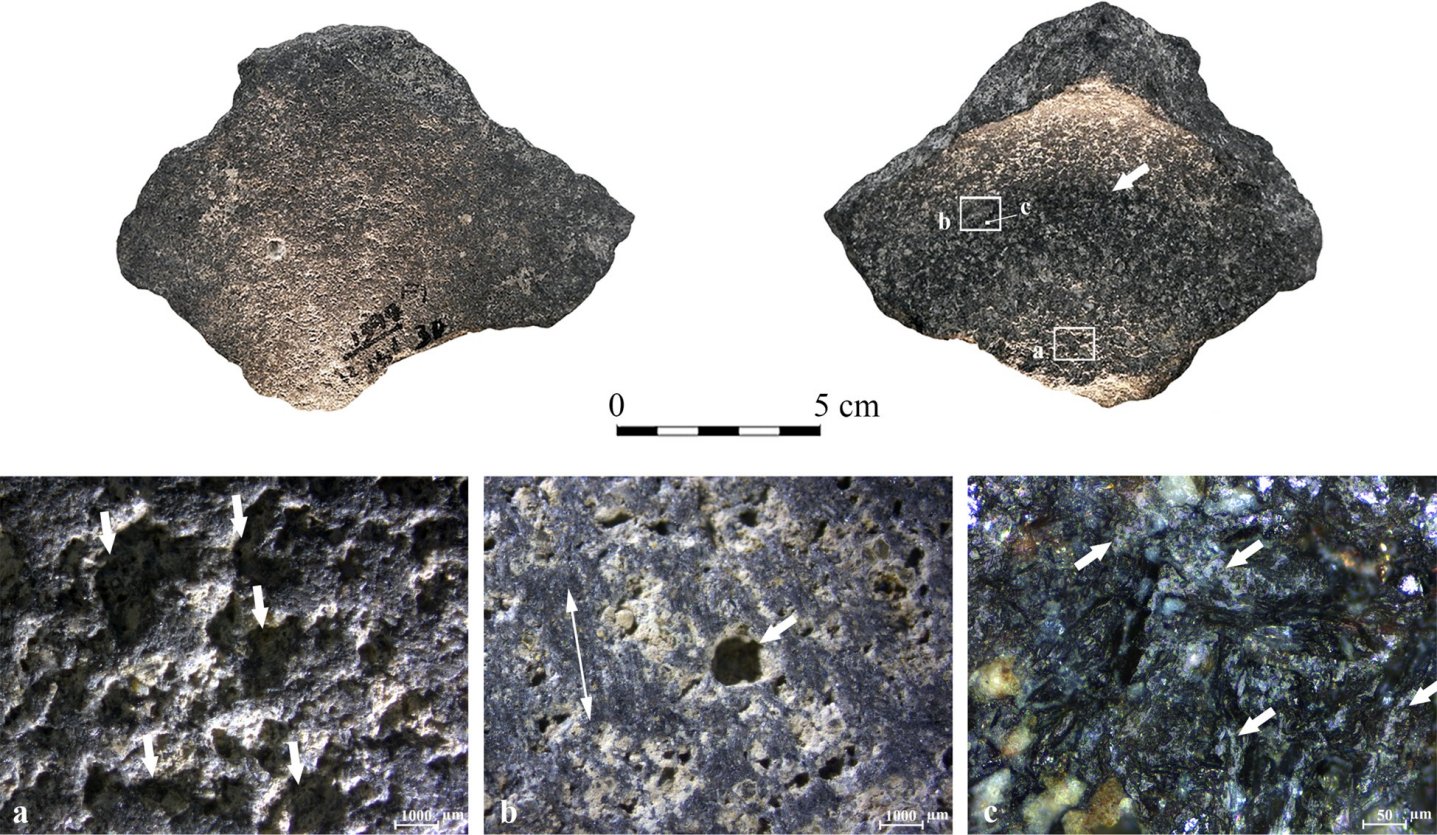
Archaeological vessel TBY37 from the Early Bronze Age I of Tel Bet Yerah, with surface darkening marked by an arrow. (a) Clear pecking marks on the elevated center shown by arrows (10x); (b) Flat leveled striated surface of the circumferential depression with a pit with sharp edges marked by an arrow. The orientation of the striations is marked by a double-headed arrow (10x); (c) A surface showing polished surfaces marked by arrows, exhibiting a reticular pattern (200x).

**TBY37** (Fig 12): the Early Bronze Age vessel is relatively small, with about one third of the base and a minor part of the wall preserved. The circumferential depression is rather wide and deep (Fig 7). In comparison to the central elevated base where clear pecking marks are observed (Fig 12A), the depression exhibits massive abrasion characterized by a flat surface with darkening and striations (Fig 12B; Table 5). The polish is remarkably bright with a reticular distribution pattern (Fig 12C). This configuration implies contact with a hard material. Further, the brightness of the polish and its distribution has parallels to the lubricant experiment using sand and water (Fig 5A), suggesting that water was used as a lubricant here.

**Fig 13:**
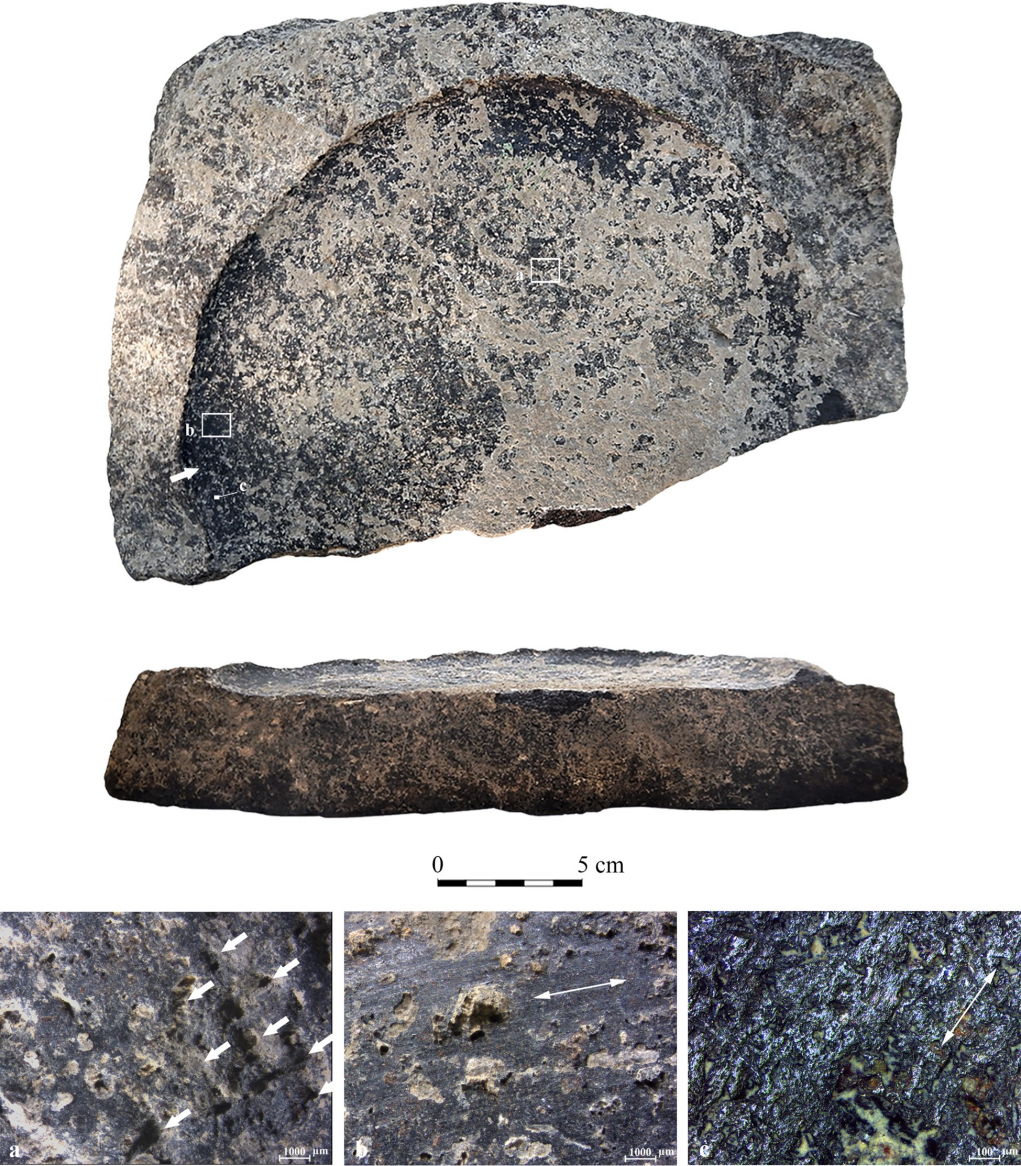
Archaeological vessel KB from Kabri, dated to the Early Bronze Age I, with surface darkening along the circumferential depression marked by a small arrow. (a) Clear pecking marks on the elevated center shown by arrows (10x); (b) Flat, leveled, striated surface of the circumferential depression with the orientation of the striations marked by a double-headed arrow (10x); (c) A heavy polish showing a reticular pattern, associated with striations in an orientation marked by a double-headed arrow along the circumferential depression (100x).

**KB** (Fig 13): the Early Bronze Age base belongs to a four-handled bowl (Fig 13). The walls are preserved to the same height (ca. 0.5 cm) along the perimeter, and the break appears relatively even, which may suggest deliberate removal [65]. The vessel is exceptionally large compared to the others, and the circumferential depression (Table 5), although relatively shallow (Fig 7), is distinct and regular. The analysis showed a remarkable difference between the wear on the central elevated base and the depression. The transition between these two areas is abrupt and very clear. The center of the base is elevated with a flat cross-section (unlike most of other pieces that exhibit a convex central base with gently sloping sides), and it is covered with pecking marks (Fig 13A shown by the arrows). Within the depression highly reflective polish and darkening are visible to the naked eye (Fig 13B). The surface inside the depression is extremely flat and striated; the parallel striations reflect a continuous rotary motion along the base’s perimeter (Fig 13B). The polish is highly developed, extremely bright, and rough, spreading in a reticular pattern (Fig 13C). It is associated with parallel micro-scale striations (Fig 13C). Based on our experiments, these traces suggest contact with a hard material, possibly involving a lubricant that would enhance the reflectivity and form the reticular polish (as in the case of the lubricant experiments with water–see Fig 6A and 6C). The wear is similar to those observed for TBY37 (described above, Fig 12).

The observations conducted on the archaeological samples in the context of the experimental results lead us to formulate several preliminary conclusions regarding production and use-wear development on the basalt vessels:

1. The appearance of the exterior and interior surfaces of the vessels is the result of their production and use-life, and unrelated to the post-deposition of the artifacts;
2. Pecking marks are the most prominent wear feature associated with the vessel manufacture, more evident on the vessel exterior wall and exterior wall-base joint, relatively less obvious on the exterior base and interior central elevated base, and virtually absent within the circumferential depression (Figs 8, 12A and 13A). It is therefore suggested that both high finishing (smoothing) and the continuous handling and use of the vessel caused the reduction of the pecking marks;
3. The experiments involving sand resulted in leveled basalt topography with rounded transitions between the plateaus and the pits and mainly superficial intermittent striations. The traces have parallels on the exterior walls, exterior bases, and central elevated bases of the archaeological vessels. Therefore, we suggest that sand was used for surface abrasion in the final stage of the vessel production;
4. The wear observed within all the circumferential depressions is analogous: characterized by substantial reduction of the basalt matrix (surface darkening), leveled topography with pronounced plateaus, continuous, parallel striations (Figs 9B, 9C, 10B, 11B, 12B and 13B) covering the high topography (plateaus) and distributed along the depression, and the presence of thick, opaque, bright micropolish, in particularly pronounced cases developing into a reticular pattern (Figs 9C, 10C, 11C, 12C and 13C). The transitions between high and low topography are abrupt or slightly rounded, and the bottoms of the pits are largely unmodified.
5. The surface leveling, the presence of continuous striations, and grain edge leveling/rounding are features indicative for abrasive wear formed by friction between two surfaces (grinding). Based on the experiments (Figs 5A, 5B, 5E, 5F, 5I, 5J and 6C), the flat topography of the circumferential depressions, associated with the wear development mainly on the protruding surfaces and prominent striations are a result of contact with a hard compact material of highly abrasive properties–likely a high-asperity stone;
6. The thick, opaque, and bright micropolish within the circumferential depressions exhibits irregular-to-sinuous morphology and occasionally develops reticulation. It spreads mainly on the elevated areas in between macro-scale striations, either in isolated patches on topographic peaks or in a covering manner (Figs 9C, 10C, 11C, 12C and 13C). It suggests that while the striations are the result of a contact with an abrasive stone, the micropolish resulted from an interaction of basalt with a different type of material. Based on our experiments (Figs 5G, 5H, 5K, 5L and 6C), this type of polish results from the contact with a semi-resilient material. While the experimental results are inconclusive in this matter, we are inclined to propose the involvement of a fine mineral powder as our main suggestion. Contact with wood may also be considered as it shares some of the polish characteristics;
7. Based on our experiments (Fig 6), during the formation of circumferential depressions water was involved as a lubricant, enhancing both reticulation and reflectivity of the micropolish;
8. The wear intensity within the depressions (depth of the depression, the degree of surface leveling, the degree of micropolish development and the presence of surface darkening) differs between the vessels. It is suggested these differences are the results of varying intensity and duration of the activity that formed them;

## 4. Discussion

The increase in basalt vessel production during the Late Chalcolithic and Early Bronze Age I is widely discussed. Studies advance several themes such as provenance [e.g., 81, 84, 93, 122, 123], production [e.g., 23], distribution and context [e.g., 3, 65, 70, 91, 92], and symbolism and conventions [e.g., 18, 69]. However, the function or functions of these vessels remain largely unclear.

**Fig 14:**
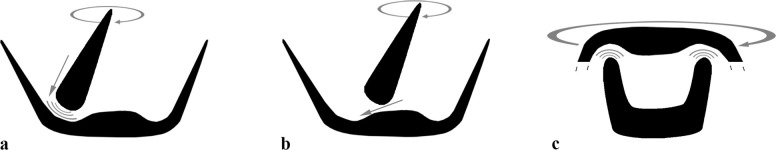
Schematic representations of the hypothetical mechanisms related to the circumferential depression. (a) Using the vessel wall as a retaining barrier for a tool while processing a material inside the vessel; (b) Restricting the tool use within the base perimeter, which facilitates the rotation and confines the distribution of the wear; (c) Forming the circumferential depression due to the vessel use as a turntable.

The results of the current study indicate that these were not just storage or serving vessels; rather, at least some of these were intensively used as a platform for a specific activity. The results clearly show that all the analyzed vessels reveal an analogous pattern of wear distribution, where wear within the circumferential depressions is significantly different from the wear on other tested areas of the vessels. These other areas–exterior base, walls, and interior central elevated base–exhibit wear traces indicative primarily of the vessel manufacture. In contrast, the wear within the circumferential depressions exhibits a distinct orientation, following the axis of the depression, and the surface is flat and highly reflective. This implies that the activity that formed these traces was limited to the base perimeter, with slight or no effect on adjacent surfaces of the interior of the vessel. Considering the dimensions of the depression and the orientation of the traces within, a narrow-ended tool (ca. 1.5–3.5 cm in width, see Fig 7) was likely used and consistently performed a rotational movement. This activity used the vessel wall as a retaining barrier for the force of a rotating device (Fig 14A), leaving a distinct channel-like depression. The experimental results indicate that the traces in the depression were formed through friction between the basalt surface and two other types of material in contact: a hard and highly abrasive stone and a semi-resilient substance like a mineral powder (e.g., stones and minerals that were processed for a paste) or perhaps wood. Furthermore, our results indicate that water was added either as a component of a worked material or an additive, which was essential to enhance the formation of the depression.

The combination of these two wear types reflects an activity where a hard rotating tool was used to process a semi-resilient material (or vice-versa, a wooden tool was used to process a highly abrasive mineral) with water. This is particularly true of four of the analyzed vessels (YH42, GHO1, TBY37, and KB), which exhibit massive matrix reduction, continuous parallel striations, and poorly altered low topography. Moreover, last three attest for abrupt pit edges (Figs 11B, 12B and 13B), grain leveling (see Table 5), and surface darkening (Figs 11–13, shown by white arrows on the macro-photographs), which are typical of intensively abraded surfaces.

One vessel shows evidence for unidirectional rotation (GHO1, Fig 11B), exhibiting comet-shaped pits, while others may have been worked bidirectionally, suggesting alternating motion of the rotating device. Notably, one archaeological sample (YH9, Fig 9) displays a prominent surface rounding with minor reduction of the basalt matrix, finer striations evident only under high magnification, and additional type of thin, translucent polish spreading within lower topographic areas (Fig 9C). The indications of rotational abrasive activity, however less pronounced, are still present in this sample; it is suggested that the rotating tool was likely made of more pliable material than in the other examples, exhibiting lower asperity. This would also explain the superficial nature of the depression in comparison to the other tested vessels (Fig 7A).

Looking at the characteristics of the wear, the results allow us to address the general functionality of the basalt vessels. In terms of context, the circumferential depression is not a widespread feature, appearing inconsistently on a small fraction of the Late Chalcolithic and Early Bronze Age vessels, with no association to a particular vessel type and size or geographic terrain. We acknowledge that context alone is a poor indicator of vessel function, especially when discussing vessel fragments. However, because basalt bowls are often assigned a prestigious value and ritualistic function based on their presence in mortuary assemblages, we consider it important to mention that the majority of basalt vessels and fragments were discarded within settlements–associated with domestic activity layers or deposited in subterranean complexes of unknown function [e.g., 97], and some show signs of intensive utilization. Many were uncovered inside shafts and waste pits located within or nearby the habitation areas of settlements [e.g., 23, 124, 125], which are often filled with debris characteristic of domestic activities–pottery fragments, animal bones etc. The vessels are mostly found broken, and there is no clear link between these fragments and food processing or storage.

This contextual variation and the variation of the traces in the basalt vessels suggest that the vessels were used for an array of functions. For example, vessels are traditionally viewed as part of the food processing industry, and although the current study cannot definitively link basalt vessels to the processing of staple crops characteristic of the Late Chalcolithic and Early Bronze Age–cereals, legumes, and olives [e.g., 11, 12, 15, 46], it is possible that the analysis of other vessels may reveal connections to food processing. Indeed, there are few examples where the basalt vessels occur alongside food processing and storage apparatuses, like grinding stones, pestles, cooking installations, pots, and storage jars [e.g., 95, 126, 127]. This includes one example of a four-handled basalt vessel fragment with a circumferential depression that was found *in situ* in association with several grinding slabs, a pestle, and multiple fragments of large storage and cooking vessels and small serving bowls [see Fig 39:1 in 128].

The dimensions of the depression reflect, in part, the dimensions of a pestle or any other tool that was operated in these vessels. However, the relatively rare Late Chalcolithic and Early Bronze Age pestles [e.g., 61, 129, 130] are all much wider than the identified circumferential depressions, so they could not act as the tools that created them. The lack of stone pestles in the assemblages and their incompatibility combined with evidence for activities related to abrasion, crushing, and rotary movements in many limestone and basalt vessels drive scholars to suggest that wooden pestles were used [e.g., 77]. Based on the use-wear analysis (Fig 5G and 5H), wood might potentially be one of the materials involved in forming the polish inside the circumferential depressions, but evidence for this is so far insufficient.

Alternatively, the analysis suggests that at least some of the basalt vessels were used to process materials unrelated to food in various craft industries. This is partially supported by contextual data. Basalt vessels *in situ* were occasionally found in a direct association with copper smelting installations and waste (e.g., Ashqelon Afridar, Ashqelon Barnea [40, 61, 96, 131]) and it has been suggested they were used for the copper ore processing [61, 131]. The example from Ashqelon Barnea includes one complete flaring bowl likely bearing a circumferential depression [see Fig 5:8 in 61]. In addition, some vessels may reveal evidence for pigment processing (with one published example bearing residue of a red pigment [77]). Pigments–especially red-brownish ones–were used for decorative purposes during the Late Chalcolithic and Early Bronze Age periods, in particular for painting pottery vessels, figurines, and ossuaries, and occasionally–during the Chalcolithic period–wall paintings [e.g., 16, 132–134].

The consistent character of the rotary phenomenon suggests that the vessels were used in an activity involving repetitive turning or stirring where wear features accumulated along the course of the depression. A related possibility views the circumferential depression as a type of intentional vessel design. It could be roughly formed in the production stage to facilitate a secure rotational movement, which would be especially helpful when working with a lubricant or a powder. The pre-formed depression would help restrict the tool’s movement to the circumference and would explain the confined distribution of the wear (Fig 14B). It would also allow the processed material to accumulate with ease in the base perimeter. The majority of the circumferential depressions are merely a few millimeters deep (see Fig 7), so their formation could also result from use alone; however, pre-shaping would explain the peculiar regularity of the wear. It is important to emphasize though that even if the channel-like features were present before vessel use, the wear within them was formed entirely during further extensive and repetitive use that also certainly impacted shape and depth of the depressions.

The wear characteristics may relate to different crafts that required processing hard materials with the addition of water, such as minerals and clay. The closest example of similar micropolish comes from the stone abrader found in the Natufian mud-plastered grave at the site of Hilazon Tachtit [Fig 10 in 113, 135]. According to the authors, this type of wear was created from abrasion against a flexible yet rough surface like leather-hard clay (in this case mud-plastering), and it was compared to traces identified on pebbles used for pottery burnishing [136]. Considering this, the use-wear identified in the circumferential depression may be in some way related to clay/pottery manufacture.

The pottery industry during the Late Chalcolithic and particularly during the Early Bronze Age advanced with typo-morphological standardization, specialized production in workshops, and interregional distribution [e.g., 3, 16, 19, 20, 137, 138]. Partially tournette-formed ceramic vessels [139–141] were attested already in the Late Chalcolithic period, and their number increased significantly during the Early Bronze Age [61, 139, 141 and references therein]. Still, it was not until the Early Bronze Age II when potter’s wheels appeared in significant frequencies [e.g., 137, 141, 142].

An interesting related, yet still unsupported suggestion, is that the basalt vessels with circumferential depressions were involved in the pottery industry, used for rotating a vessel while coiling/shaping. The circumferential depression would be then formed by using the vessel base as a turntable on top of a smaller vessel or a narrow socket made of a hard abrasive material (i.e., the rim creating the circumferential depression while rotating; Fig 14C), respectively in place of upper and lower potter’s wheels, which are conspicuously missing from the Chalcolithic material culture repertoire. Similar suggestion was made by Rosenberg [143] in relation to a perforated basalt vessel base from Ashqelon Barnea. This would explain the uncommon nature of the circumferential depressions within the vessels versus their broad geographic distribution across the Late Chalcolithic and Early Bronze Age southern Levant.

To conclude, it should be stressed that all of the tasks mentioned above relate to a processing activity that required rotary movement and perhaps benefited from the interaction between the ‘active’ tool and the vessel. The rotation was conducted with a high level of consistency and regularity, and it was probably enhanced by water and/or an intermediate material. Based on the characteristics of the depressions and wear, this action was likely achieved using a compact and narrow abrasive object. While minor differences in the tools used or the materials processed may exist, the same phenomenon was applied to all vessels. This activity was performed in typologically varied basalt vessels, starting during the Late Chalcolithic and continuing through the Early Bronze Age. Considering this, these specific basalt vessels provide evidence for a direct techno-functional link between these two periods in the southern Levant, showing that concepts or traditions of vessel use were shared for a long period of time.
